# SPICT as a predictive tool for risk of 1-year health degradation and death in older patients admitted to the emergency department: a bicentric cohort study in Belgium

**DOI:** 10.1186/s12904-023-01201-9

**Published:** 2023-06-24

**Authors:** Delphine Bourmorck, Marie de Saint-Hubert, Marianne Desmedt, Ruth Piers, Julien Flament, Isabelle De Brauwer

**Affiliations:** 1 Institut de Recherche Santé et Société, Université catholique de Louvain (UCLouvain), Clos Chapelle-aux-Champs, 30, Bruxelles, 1200 Belgium; 2 Centre Hospitalier Universitaire - UCL - Namur, Avenue Gaston Thérasse 1, Yvoir, 5530 Belgium; 3 Cliniques universitaires Saint-Luc, Avenue Hippocrate 10, Bruxelles, 1200 Belgium; 4 Department of Geriatrics, Ghent University Hospital, C. Heymanslaan 10, Ghent, Gent, 9000 Belgium

**Keywords:** SPICT, Palliative Care, Emergency Department, Prognosis, Older patients

## Abstract

**Background:**

Older patients are increasingly showing multi-comorbidities, including advanced chronic diseases. When admitted to the emergency department (ED), the decision to pursue life-prolonging treatments or to initiate a palliative care approach is a challenge for clinicians. We test for the first time the diagnostic accuracy of the Supportive and Palliative Care Indicators Tool (SPICT) in the ED to identify older patients at risk of deteriorating and dying, and timely address palliative care needs.

**Methods:**

We conducted a prospective bicentric cohort study on 352 older patients (≥ 75 years) admitted to two EDs in Belgium between December 2019 and March 2020 and between August and November 2020. SPICT (French version, 2019) variables were collected during the patients’ admission to the ED, along with socio-demographic, medical and functional data. The palliative profile was defined as a positive SPICT assessment. Survival, symptoms and health degradation (≥ 1 point in ADL Katz score or institutionalisation and death) were followed at 12 months by phone. Main accuracy measures were sensitivity, specificity and likelihood ratios (LR) as well as cox regression, survival analysis using the Kaplan Meier method, and ordinal regression.

**Results:**

Out of 352 patients included in the study (mean age 83 ± 5.5 years, 43% male), 167 patients (47%) had a positive SPICT profile. At one year follow up, SPICT positive patients presented significantly more health degradation (72%) compared with SPICT negative patients (35%, p < 0.001). SPICT positivity was correlated with 1-year health degradation (OR 4.9; p < 0.001). The sensitivity and specificity of SPICT to predict health degradation were 0.65 (95%CI, 0.57–0.73) and 0.72 (95%CI, 0.64–0.80) respectively, with a negative LR of 0.48 (95%CI, 0.38–0.60) and a positive LR of 2.37 (1.78–3.16). The survival time was shorter in SPICT positive patients than in SPICT negative ones (p < 0.001), the former having a higher 1-year mortality rate (HR = 4.21; p < 0.001).

**Conclusions:**

SPICT successfully identifies older patients at high risk of health degradation and death. It can support emergency clinicians to identify older patients with a palliative profile and subsequently initiate a palliative care approach with a discussion on goals of care.

## Background

The benefits of a palliative care approach are well known. It increases patients’ chance of passing away in the comfort of their own home, improves the quality of life and alleviates burdensome symptoms such as depressive symptoms and pain [1–6]. Patients receive less aggressive end-of-life care, make less use of the emergency department (ED) and have fewer acute hospitalisations [1, 3, 6–10]. A palliative care approach may consider life prolonging treatments and takes into account the patient’s comfort and care wishes. However, palliative care approaches are still rarely and lately implemented in Belgium [11, 12]. Emergency departments could have a role in earlier introduction of a palliative care approach as the admission in ED in the last year of life is high, reaching 30–75% in some countries [13–16].

Up to a quarter of ED patients are aged 65 and over [17]. Older patients in the ED often present a profile of multi-comorbidity, chronic diseases, frailty, reduction in daily activities and burdensome symptoms [18–20]. Some of these older patients may present a palliative care profile, defined by 3 distinct clinical situations: (1) acute exacerbation of symptoms that may be potentially fatal (2) acute life-threatening conditions caused by external events or (3) advanced, incurable progressive diseases with indicators of frailty [21].

Initiating a palliative care approach remains a challenge in EDs. It is difficult for emergency clinicians to decide whether to pursue invasive and uncomfortable life-prolonging treatments or to reassess intensity of treatment and consider a palliative care approach [20]. For some older patients, the life prolonging treatments offered in the acute ED conditions may have a poor added value regarding the overall disease trajectory [22, 23]. In addition, they may not be aligned with goals of care and patient preferences. Yet, the medical decisions made during ED stay are crucial to determine the care trajectory.

Initiating a palliative care approach in EDs is not much studied, but presents interesting results such as shortened hospital stay [24], reduced use of intensive care and improvement of patient and family satisfaction [25]. Indeed, patients who benefit from a palliative care approach return home more quickly, or are more easily referred to outpatient palliative care [24, 25].

However, a major challenge in implementing a palliative care approach is to identify which patients might benefit from such care. There is a lack of consensus about the illness stage from which it is advisable to initiate palliative care for each specific disease [26]. Transitioning to palliative care earlier than at the very end of life remains a major issue for clinicians [26, 27].ED clinicians take care of the patients during acute events and Advanced Care Plan (ACP) documents are seldom available in the patients’ file, which is not conducive for in-depth discussion about care during ED stay [28]. To be able to decide whether to pursue life-prolonging treatments or to initiate a palliative care approach, ED clinicians must be supported in the identification of older patients who can benefit from a palliative care approach.

Several tools aim to identify patients who might benefit from a palliative care approach [29, 30]. The Supportive and Palliative Care Indicators Tool (SPICT) has some features recommended for use in ED, such as its speed of completion, its possible use by triage nurses and its consideration of all types of diseases [25]. The SPICT is composed of general indicators of health deterioration as well as disease severity indicators. SPICT was developed in the United Kingdom to help healthcare professionals identify patients at risk of deterioration and death and support them to timely initiate palliative care [21]. This tool could support ED clinicians identify older patients with a palliative profile and address palliative care as care approach. However, it has not yet been tested in ED for older persons (≥ 75 years).

The aim of this study is to assess the SPICT accuracy to predict 1-year health degradation and death in older patients admitted to ED.

## Methods

The paper is reported following the STROBE statement.

### AIM

The aim of the study is to assess the SPICT (2019, French version) accuracy to predict 1-year health degradation and mortality while used during an ED admission of older patients aged 75 years old or more. The SPICT is a tool composed of 6 general indicators of health deterioration and 23 indicators of severity of specific-diseases which aim at identifying patients with a palliative profile [21]. SPICT assessment is positive when at least one general and one disease specific indicators are identified [21]. SPICT is available on www.spict.org.uk. Permission to use it was obtained.

### Design

We conducted a prospective cohort study in the emergency department of two tertiary Belgian hospitals between end-December 2019 and November 2020. The patient’s inclusion lasted 6 months in total, with an interruption between mid-March to mid-August 2020 due to the Covid-19 pandemic.

### Participants

All French speaking patients of 75 years old or more admitted to ED were eligible for the study. Patients unable to give consent were included if their legal representative approved the participation. We excluded dying patients or patients previously included in the study. Dying patients were defined as likely to die within 24 h following admission to the ED.

### Data collection

Three researchers collected data during office hours (8 am to 6 pm). Training and supervision on data collection was performed before and during the whole period to ensure the quality and homogeneity of data collection. The supervision was realised by the main investigator who is a geriatrician (IDB) and included weekly discussions on patient inclusion and random checks of SPICT completion based on medical records.

The researchers carried out face-to-face interviews with the patient or their legal representative during ED admission to obtain data on demographic, psycho-social characteristics, functional status using Katz [31], Lawton [32], and the general poor health indicators of the SPICT [21]. For the Katz and SPICT items, the patient or their legal representative had to refer to the situation two weeks before admission [33]. Data was also collected through the medical file at the patient discharge, i.e. principal diagnostic at ED with ICD10 classification [34], number and type of comorbidities encoded according to the 16 medical diagnoses listed in the Charlson Comorbidity Index [35], length of ED stay, survival, place of discharge, availability of an advance care plan (ACP) or any reference to wishes about end-of-life care, availability of a “do not resuscitate” code (DNR code) or any treatment limitation, and clinical indicators of life limiting conditions of the SPICT [21]. SPICT results were not communicated to the regular healthcare providers in order to avoid bias in usual care and decision making.

From the beginning of the COVID-19 pandemic, we did not include patients admitted in COVID-area of the ED for practical and security reasons.

The principal researcher (DB) followed-up patients by phone at 12 months after ED admission. Data collected in the follow-up calls were mortality (date, place of death, and if death occurred in a COVID-19 positive context), functional status (Katz), place of residence in the last 3 months of life, number of readmissions (ED, hospital), palliative care in the last 3 months and symptoms (Symptom Assessment scale) [36].

### Outcomes

The main outcome measure is health degradation. Health degradation is a composite outcome that intends to be more comprehensive than mortality alone, as mortality has its limits in justifying a palliative care approach. Health degradation combines the Katz score, the institutionalisation in a nursing home and mortality. Health degradation is a categorical variable with three levels. Score 1 corresponds to an improvement or constancy in the person’s health status (no decrease in the Katz score, no institutionalisation, no death). Score 2 corresponds to a decrease in functional capacity according to the Katz scale (loss of at least 1 point out of a total of 6) or to a patient being institutionalized. Score 3 corresponds to death.

The palliative profile was defined by a positive SPICT assessment, i.e. when at least one general and one disease specific indicator of the SPICT were identified [21]. The indicator “unplanned hospital admission” of the SPICT was positive if the patient stayed at least one night in the hospital after ED admission.

### Statistical analysis

Continuous variables were described using mean and standard deviation. Categorical variables were described using the number and their percentage. Comparisons of patient characteristics, between patients with or without a palliative profile, were assessed with t-test for continuous variables and Chi-squared for categorical variables. Symptoms at 1 year after ED admission were compared between groups with and without a palliative profile with a non-parametric median test for independent groups, corrected for Yates continuity.

The accuracy of SPICT to predict 1-year health degradation and death is assessed by sensitivity, specificity, likelihood ratio, positive and negative predictive values and the overall accuracy (proportion correctly classified). To perform these analyses, the variable “health degradation” was binarised, i.e., no health degradation (0 = score 1) and health degradation (1 = score 2 or 3). Association between SPICT and health degradation (3 levels variable) was assessed through a 3-level ordinal regression, adjusted for age (continuous variable) and gender (categorical variable). A multi-variable cox-regression was used to assess the association between SPICT and 1-year mortality, also adjusted for age (continuous variable) and gender (categorical variable). A survival analysis was performed on mortality using Kaplan Meier method.

The statistical analyses were computed using IBM SPSS software V27 and NCSS 2021.

### Ethical approval

The Ethical Committees of the two participating hospitals gave their agreement. The study registered number is B403201941609. Informed consent was signed before any data collection by the patients and/or their legal representative.

## Results

**Fig. 1:**
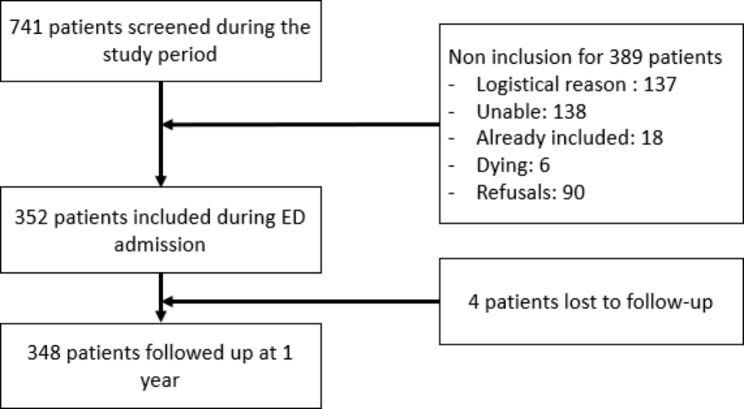
Patient inclusion flowchart *logistical reason: lack of time or availability for the researcher to include the patients

The flowchart for the inclusion of patients in the study is presented in Fig. 1. Included patients represented 47.5% of the screened population.

### Population characteristics and 1-year mortality

The mean age of our participants was 83 years old. Of the 352 patients included, 43% (n = 150) were male and 49% (n = 172) were living in partnership. Wishes for end-of-life care or DNR code was known for 2.3% (n = 8). Results of advance care planning weren’t available for any patient. The main diagnoses (ICD-10CM chapters) at admission to ED were ‘abnormal signs’ (23%), circulatory system (19%), injury and other consequences of external causes (15%) and digestive system (9%) (Table 1).

**Table 1:** Characteristics of the study population according to SPICT assessment

	Total n = 352	SPICT- n = 185	SPICT+ n = 167	p-value
	n (%) or mean (± SD)	n (%) or mean (± SD)	n (%) or mean (± SD)	
Sociodemographics Gender, male Mean Age, years Age, 85+ Being in partnership Living in Nursing home Living at Home Primary education or less Receiving formal or informal care	150 (42.6) 83.2 (± 5.5) 143 (40.6) 172 (49.0) 24 (6.8) 318 (90.3) 36 (10.3) 302 (85.8)	78 (42.2) 82.5 (± 5.6) 64 (34.6) 96 (51.9) 4 (2.2) 177 (95.7) 17 (9.2) 152 (82.2)	72 (43.1) 84.0 (± 5.2) 79 (47.3) 76 (45.8) 20 (12.0) 141 (84.4) 19 (11.5) 150 (89.8)	0.86 0.008 0.015 0.25 0.000 0.000 0.48 0.04
**Functional characteristics** ADL total score 2 weeks before admission in ED iADL total score 2 weeks before admission in ED Falls during previous year	1.4 (± 0.6) 4.4 (± 2.1) 159 (45.4)	1.2 (± 0.2) 5.4 (± 1.6) 70 (38.3)	1.7 (± 0.7) 3.3 (± 2.0) 89 (53.3)	0.000 0.000 0.005
**Principal diagnosis at admission** Abnormal signs, Chap. 18* Circulatory system, Chap. 9* Injury and other consequences of external causes, Chap. 19* Digestive system, Chap. 11* **Comorbidities** (≥ 2 diseases) **Medication**	81 (23.0) 67 (19.0) 52 (14.8) 33 (9.4) 170 (48.6) 7.2 (± 4.1)	41 (22.2) 29 (15.7) 33 (17.8) 22 (11.9) 66 (36.1) 6.0 (± 3.4)	40 (24.0) 38 (22.8) 19 (11.4) 11 (6.6) 104 (62.3) 8.4(± 4.4)	0.69 0.09 0.08 0.08 0.000 0.000
**Hospitalised** Medical Geriatric Surgical Middle care Stroke unite ICU Palliative	169 (48.0) 76 (45.0) 51 (30.2) 23 (13.6) 10 (5.9) 9 (5.3) 0	57 (30.8) 32 (56.1) 6 (10.5) 12 (21.1) 5 (8.8) 2 (3.5) 0	112 (67.1) 44 (39.3) 45 (40.2) 11 (9.8) 5 (4.5) 7 (6.3) 0	0.000
**Wishes for end-of-life care known** **Availability results of ACP at the ED** **Treatment limitation during ED stay**	8 (2.3) 0 (0.0) 11 (3.1)	0 (0.0) 0 (0.0) 1 (0.5)	8 (4.8) 0 (0.0) 10 (6.0)	0.003 - 0.003
**6 months mortality** **1-year mortality**	59 (17.4) 75 (22.7)	11 (6.1) 17 (9.8)	48 (30.0) 58 (36.9)	0.000 0.000
**Health degradation after 1 year** No degradation Degradation Death	(n = 304) 145 (47.7) 84 (27.6) 75 (24.7)	(n =160) 105 (65.6) 38 (23.8) 17 (10.6)	(n = 144) 40 (27.8) 46 (31.9) 58 (40.3)	0.000

*Principal diagnosis at admission using the ICD10 classification, ADL: Activity of daily living, range 0–6, high score = high dependency [31], iADL: instrumental activity of daily living, range 0–7 high score = independency [32], p-value assessed by t-test for continuous variables, Chi-squared test for categorical variables

### SPICT criteria and palliative profile

**Fig. 2:**
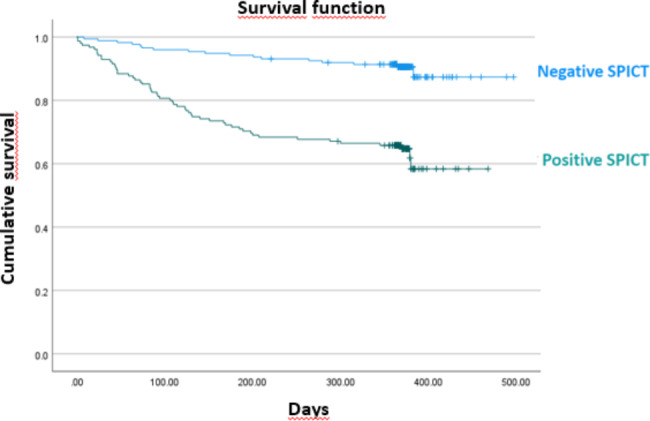
Survival Analysis, Kaplan Meier Log Rank test = p < 0.001

Almost half of the patients included (47.4%) had a palliative profile, i.e. a positive SPICT assessment. Patients with a palliative profile were significantly older (mean 84 vs. 82.5 years, p = 0.008) with a higher proportion of patients aged 85 or more (47.3% vs. 34.6%, p = 0.015). They more often lived in nursing homes (20% vs. 4%, p < 0.001) and received more formal or informal care (86% vs. 82%, p = 0.04). Patients with a palliative profile were more dependant for daily activities (Katz score: 10 vs. 7, p < 0.001) and for the instrumental activities of daily living (iADL) (Lawton score: 3 vs. 5, p < 0.001) which means their functional status was poorer. They had more often comorbidities (62% vs. 36%, p < 0.001) and used more medications (mean number 8 vs. 6 medications, p < 0.001). During ED admission, patients with a palliative profile got more treatment limitations (0.5% vs. 6%, p = 0.003). After ED admission, patients with a palliative profile were more often hospitalised (67% vs. 31%, p < 0.001), and died significantly more at 6-month (30% vs. 6%, p < 0.001) and 12-month (37% vs. 10%, p < 0.001) (Table 1; Fig. 2).

### Prognosis accuracy of SPICT and 1-year outcome

Table 2 shows the main parameters of the SPICT accuracy to predict 1-year health degradation (first column) and death (second column).

**Table 2:** Description of the SPICT accuracy to predict 1-year health degradation and death

	Health degradation Total (n = 304) Value (95% CI)	Death Total (n = 298) Value (95% CI)
SPICT sensitivity	0.65 (0.57–0.73)	0.77 (0.66–0.86)
SPICT specificity	0.72 (0.64–0.80)	0.61 (0.55–0.67)
Overall Accuracy	0.69 (0.63–0.74)	0.65 (0.60–0.70)
Diagnostic Odds Ratio	4.96 (3.01–8.10)	5.41 (2.98–9.82)
Positive Predictive Value	0.72 (0.64–0.79)	0.37 (0.29–0.45)
Negative Predictive Value	0.66 (0.58–0.73)	0.90 (0.85 − 0.74)
Negative Likelihood Odds Ratio	0.48 (0.38–0.60)	0.37 (0.24–0.57)
Positive Likelihood Odds Ratio	2.37 (1.78–3.16)	2.00 (1.64–2.43)

The SPICT overall accuracy reached 0.69 and a diagnostic odds ratio of 4.96. The sensitivity and specificity were respectively 0.65 and 0.72. One-year positive and negative predictive values were 0.72 and 0.66. SPICT informative values were moderate with a positive and negative likelihood ratio of 2.37 and 0.48. Those results showed a moderate accuracy of the SPICT for health degradation at 1 year (Table 2).

### One-year survival analysis

The ordinal regression, adjusted for gender and age, showed that a palliative profile as defined by a SPICT positive test was positively associated with 1-year health degradation (OR 4.9; p < 0.001) (Table 3).

**Table 3:** Ordinal regression for health degradation at 1 year after ED discharge

	OR (95% CI)	p-value
**SPICT**	4.9 (3.10–7.77)	0.000
Age	1.06 (1.02–1.11)	0.003
Gender, male	1.38 (0.88–2.16)	0.16

The cox-regression model, adjusted for gender and age, showed that mortality was associated with older patients having a palliative profile (HR = 4.2; p < 0.001) (Table 4). Survival time was shorter in older patients with such a palliative profile (χ^2^ = 34.0, p < 0.001) (Fig. 2).

**Table 4:** Cox regression for mortality at 1 year after ED discharge

	HR (95% CI)	p-value
**SPICT**	4.21 (2.43–7.27)	0.000
Age	1.03 (0.99–1.08)	0.01
Gender, male	1.52 (0.96–2.40)	0.08

At 1-year follow-up, older patients with a palliative profile reported significantly higher symptoms related to appetite, fatigue, confusion and swallowing difficulties compared to patients without a palliative profile (Table 5).

**Table 5:** Palliatives indicators at one-year follow-up for the survival patients

	1 year follow-up (n = 231)		
	SPICT- median, SD (n)	SPICT+ median, SD (n)	global median; p-value*
**Breathlessness**	3, 2.5 (n = 144)	4.5,2.9 (n = 86)	4; 0.11
**Depressed feeling**	2, 2.6 (n = 144)	3, 3.0 (n = 84)	2; 0.19
**Feeling nervous**	3, 2.7 (n = 145)	3, 2.6 (n = 86)	3; 0.72
**Pain**	3, 2.7 (n = 145)	4, 3.0 (n = 85)	3.5; 0.79
**Respiratory secretions**	0, 2.3 (n = 144)	0, 2.8 (n = 84)	0; 0.48
**Swallowing problems**	0, 2.1 (n = 145)	0, 2.4 (n = 86)	0; 0.03
**Lack of appetite**	0, 2.7 (n = 145)	3, 3.1 (n = 86)	0; 0.04
**Fatigue**	5, 2.3 (n = 144)	5, 2.6 (n = 86)	5; 0.002
**Confusion**	1, 2.6 (n = 145)	3, 3.3 (n = 86)	2; 0.03
**Lack of muscular energy**	4, 2.4 (n = 145)	5, 2.4 (n = 85)	5; 0.07

*Non-parametric median test with Yates correction

Numeric scores between zero (no difficulties) to ten (highest difficulties, can’t be worse), assessed by patients

## Discussion

To our knowledge, this is the first prospective cohort study which assesses SPICT in emergency department and uses health degradation as main outcome measure.

### Main findings

The aim of this study is to assess the accuracy of the SPICT in predicting health degradation and death of older patients who are admitted to ED. Almost half of the patients in this cohort were positively assessed with SPICT, indicating they have a palliative profile. In the year following ED admission, 37% of the patients identified with a palliative profile died (vs. 10% for patients without a palliative profile) and they presented significantly more health degradation (72%) compared the non-palliatives ones (35%, p < 0.001). Most deaths occurred in the first 6 months after the ED admission. The odds ratio of health degradation was 5 times greater and the hazard ratio of death was 4 times greater for older patients with a palliative profile compared to the non-palliative ones. The results of the diagnostic accuracy of the SPICT were moderate in predicting 1-year health degradation (AUC: 0.69) and death (AUC: 0.65). Our preliminary analyses showed that using health degradation instead of death improved slightly the overall accuracy of the SPICT, the specificity (0.72 vs. 0.61), the positive LR (2.37 vs. 2.00) and the positive predictive value (0.72 vs. 0.37). However, it reduces the sensitivity (0.65 vs. 0.77) (included in Table 2).

### Discussion of the results

There is limited evidence on diagnostic accuracy of tools to predict palliative care needs within ED. A recent literature review identified and compared screening tools to identify patients with palliative care needs in ED [37]. The SPICT was not included as it was not yet assessed in ED. The main tool used was the Surprise Question (SQ: “Would you be surprised if your patient died in the next 12 months?“). The overall accuracy of the SQ in predicting death from the ED is similar to our results for SPICT (QS : Area under the curve (AUC) 0.67). Although our sensitivity is slightly higher and our specificity lower (QS: sensitivity 0.63, specificity 0.75) [37]. These differences could be explained by the internal construction of the two tools: the surprise question is based on medical intuition which relies mainly on indicators of disease severity whereas the SPICT refines his sensitivity through general health indicators [21, 38].

The SPICT accuracy to predict death was assessed within different care settings for older patients and their results differ slightly from ours. The overall accuracy of the SPICT during hospitalisation on geriatric wards had higher general accuracy and sensitivity but lower specificity than our results in ED (Area under the curve (AUC) 0.82, sensitivity 0.82, specificity 0.49 [39]; AUC 0.76, sensitivity 0.84, specificity 0.58) [40]. These differences may be explained by the context of care, where clinicians care for older patients over a longer period of time which contributes to a better identification of the different health indicators. Another explanation is the different cut-off used for the SPICT by De Bock et al., who used two general indicators to obtain a positive SPICT assessment while we used only one [40]. Nonetheless, SPICT usually presents a greater sensitivity than specificity to predict death, alike the results of our study.

### Clinical implications

The main objective of the SPICT is to support clinician to early identify patient at risk of health degradation and death, who may benefit from palliative care approach, which is a major difficulty for clinicians (41). In our study we found that patients with a positive SPICT assessment have 72% risk of health degradation within the year. The overall accuracy of 69% to predict health degradation is satisfying. In addition, the SPICT positive patients surviving after one year have exacerbated symptoms compared to SPICT negative patients. These elements should alert clinicians to think about palliative care and adapt their routine of care in ED. The early identification of patients who might need palliative care is important for patients who will gradually present health degradation and die in the next months. SPICT can sensitise the ED clinicians on early palliative care which is not reserved for the very end-of-life (last days or weeks of life) and which is also appropriate for other chronic conditions than cancerous diseases [27]. When a palliative patient profile is identified, it’s the right moment to take time, to think about potential other care trajectories, to initiate discussion about goals, wishes and preferences of care, including Advance Care Planning discussion [21]. This is important in a context where ACP is never available, even for patients coming from nursing homes. Still we are missing guidelines on what care model to follow after the identification of a palliative patient in the emergency department [24].

### Strengths and limits

A strength of our study is our main outcome measure “health degradation” and the symptoms assessment during follow-up, while most studies on the SPICT measures death as main outcome. In testing SPICT within EDs, we tried to conciliate two “a priori” opposite logics of care, namely palliative care and urgent-life sustaining care. Research on palliative care in ED and their guidelines are quite rare and recent (24,25,42).

The impact of covid-related deaths was not assessable because the cause of death was missing in 36% of the patients due to the follow-up by phone calls. For the other patients, covid was incriminated in 11% of cases (data not shown).

Our cohort of old patients admitted in ED may differ from the entire population. We included patients during office hours, we collected data on patients we could not include during our shift. We do not have information about the patient’s characteristics outside office hours.

## Conclusions

SPICT successfully identifies older patients at high risk of health degradation and death within one year. SPICT could be an interesting tool to support emergency clinicians to identify older patients with a palliative profile and subsequently initiate a palliative care approach with a discussion on the goals of care. Further research is needed to assess the feasibility of implementing SPICT and the ED’s role in early introduction of a palliative care approach.

## Data Availability

Data are available on reasonable request to the corresponding author.

## List of abbreviations

ACP: Advance Care Planning
ADL: Activity of daily living
DNR: Do Not Resuscitate code
ED: Emergency Department
iADL: Instrumental activity of daily living
SPICT: Supportive and Palliative Indicators Care Tool
